# A systematic review of effectiveness and safety of some herbal compounds as treatment for primary insomnia

**DOI:** 10.1192/j.eurpsy.2024.1608

**Published:** 2024-08-27

**Authors:** M. Barbosa, A. R. Fonseca

**Affiliations:** SPSM, Centro Hospitalar de Leiria, Leiria, Portugal

## Abstract

**Introduction:**

Sleep related disorders affect around 30% of people all over the world, and evidence shows that 10% require therapeutic intervention. Insomnia represents the most common disturbance of sleep, defined as the experience of poor sleep for at least 1 month. Most of primary insomnia can be prevented by a proper lifestyle and sleep hygiene rules. Regardless, hypnotic drugs and widely prescribed, and most times, long-term used, which is not recommended because of its negative side effects.

**Objectives:**

Review the scientific evidence about effectiveness of plant extracts for insomnia, natural products with practically no side-effects, and thus be possible to reduce or even avoid the use of hypnotic drugs.

**Methods:**

The Medline database through the Pubmed search engine was used with the following keywords: “*insomnia*” and “*herbal compounds*”.

**Results:**

Valerian activity on sleep disturbances has been attributed to the presence of isovaleric acids and valepotriates with reported calming action and GABA reuptake inhibition with sedative effects. Considering the data presented in the literature, despite controversial and conflicting, several studies showed that valerian (160-600mg/day) improved sleep quality and reduced sleep latency and duration; also valerian seems more effective for chronic insomnia than acute episodes.

Hop has different properties: calming, sleep inducing, gastric secretion stimulating and spasmolytic.

Increasing GABAergic activity seems to be the main mechanism of action, thus inhibiting the central nervous system and also has demonstrated binding affinities to some of the melatonin and serotonin receptor. It’s sedative characteristics have been confirmed in a clinical trial in association with valerian, where sleep latency and quality were improved. However, monotherapy studies showed no relevant effectiveness in sleep.

Kava Kava plant showed promising results, in rats and humans, with decrease sleep latency, better sleep quality and recuperation after sleep. However, raised concern about its potential of hepatotoxicity.

There is also promising evidence of the lavender efficacy for sleep disorders in a wide variety of populations and diseases, it was actually mentioned to be as effective as lorazepam in adults with anxiety and sleeping problems. With studies with dose of 80mg it was observed a reduction in sleep awakenings, sleep duration and overall sleep quality and anxiety.

**Conclusions:**

There is a clear preference from the patient to natural compounds, and with almost nonexistent side effects, some herbal derivates are showed to have positive effectiveness in mild insomnia, but nonetheless much more studies on this field are needed.

**Disclosure of Interest:**

None Declared

